# Prognostic Value of Circulating Biomarkers of Fibrotic Remodeling in Arrhythmogenic Cardiomyopathy

**DOI:** 10.3390/biomedicines12112623

**Published:** 2024-11-16

**Authors:** Stephen P. Chelko

**Affiliations:** Department of Biomedical Sciences, Florida State University College of Medicine, Tallahassee, FL 32306, USA; stephen.chelko@med.fsu.edu

Arrhythmogenic cardiomyopathy (ACM) is a nonischemic, familial heart disease with a high risk of sudden cardiac death (SCD) in the pediatric population and accounts for >20% of SCDs worldwide [1,2,3,4,5]. ACM disease progression worsens in an age-dependent manner and is characterized by cardiomyocyte cell death, ventricular dysfunction, wall motion abnormalities, and arrhythmias [6,7]. Myocardial inflammation is a pathological hallmark of ACM and has been considered to be the result of both infiltrating immune cells and NFĸB-mediated transcription of inflammatory cytokines from cardiomyocytes harboring pathogenic variants in desmosomal genes [6,7,8,9,10,11]. These episodic and chronic inflammatory states, often referred to as “Hot Phases” of ACM, leads to cardiomyocyte cell death and extensive fibrotic remodeling [6,7,8,9,10,11]. Although mechanisms that culminate in fibrotic remodeling in ACM are not fully understood, unfortunately, even standard imaging techniques can lead to a misdiagnosis of myocarditis [12]. Thus, a reliable, sensitive, and non-invasive circulating biomarker could be an invaluable diagnostic tool to discriminate between myocarditis and ACM. A distinction that would afford both the clinician and the patient by providing a means to track disease progression via elevations in circulating molecules (i.e., serve as an effective prognostic biomarker).

Extensive fibrotic remodeling predisposes the heart to potentially life-threating fatal arrhythmias, such as reentrant ventricular tachycardia (VT) [13,14]. Although antiarrhythmics and implantable cardioverter defibrillators (ICDs) are mainstays in disease management, they do not provide meaningful information on the presence or extent of myocardial fibrosis. Invasive retrieval of an endomyocardial biopsy (EMB) was once routinely used as a diagnostic Task Force Criteria (TFC) of ACM, as it could determine the extent of fibrotic replacement of myocardium (i.e., <60% of residual myocytes) [15,16]. Yet, the utilization of this diagnostic technique is declining due to its inherent risk during recovery, as well as the potential to generate false-positives as a result of an EMB obtained from an unaffected region of the ventricle [17]. Alternatively, cardiac magnetic resonance imaging (MRI) is a non-invasive diagnostic imaging technique used to assess both cardiac function and the presence of fibrotic/inflammatory myocardium when late gadolinium enhancement (LGE) is employed [15,16]. Considering patients with ACM have frequent clinical follow-ups, concern is warranted with the repeated use of LGE dye in patients with comorbidities, such as liver cirrhosis and/or chronic kidney disease [18,19,20]. Alternatively, circulating biomarkers could serve as a routine, sensitive, and non-invasive technique to detect fibrotic cardiac remodeling. Moreso, it could be a potentially useful correlative tool as a biomarker of electrocardiographic (ECG) alterations and arrhythmic risk. Ultimately, this prognostic approach would eliminate any adverse events associated with the retrieval of an invasive EMB and/or LGE-mediated exacerbation of existing comorbidities.

In this Special Issue of *Biomedicines*, Van der Voorn and colleagues provide strong correlative evidence between the levels of circulating carboxy-terminal propeptide procollagen type-I (PICP) and carboxy-terminal telopeptide type-I collagen (ICTP) with cardiac function in patients with ACM [21]. Collagen is an essential component of the cardiac extracellular matrix (ECM), maintaining the mechanical and structural integrity of the heart. As such, collagen type IA (COL1A1) comprises 85% of collagen isoforms [22,23,24]. Elevated levels of PICP, the fibrillar collagenous component COL1A1, have been indexed in EMBs, the coronary sinus, and the peripheral vasculature as a biomarker of myocardial fibrosis in patients with heart failure, hypertrophic cardiomyopathy, and dilated cardiomyopathy [25,26,27]. During collagen proteasomal degradation, matrix metalloproteinases (MMPs) degrade myocardial ECM, releasing PICP and ICTP into the vasculature [25,26]. Thus, circulating levels of PICP and ICTP function as indirect markers of fibrosis-induced cardiac remodeling.

As such, in this Special Issue of *Biomedicines*—Advanced Research in Arrhythmogenic Cardiomyopathy—Van der Voorn and colleagues assessed whether circulating levels of PICP and ICTP correlated with myocardial fibrosis and cardiac function in patients with ACM [21]. Van der Voorn and colleagues provided robust data demonstrating the correlative relationship between patient proband status, TFC score, and cardiac function (e.g., %LVEF and %RVEF) with circulating levels of PICP and ICTP [21]. Unfortunately, no such correlations existed with ECG depolarization/repolarization abnormalities, such as QRS duration, the number of T-wave inversions in precordial leads V_1_–V_6_, terminal activation delay (TAD), and/or bundle branch block [21]. Although there was no such correlation between PICP and ICTP levels and ECG anomalies, these biomarkers do appear to be indispensable tools that could temporally track functional and pathological phenotypes during disease progression in patients with ACM [28,29]. For example, a recent report by Bacmeister L et al, demonstrated that patients with myocarditis displayed elevated serum levels of MMP-1 and PICP compared to patients with a myocardial infarction (MI) [30].

Considering ACM is a rare disease, it should be noted that Van der Voorn et al. were able to enroll a considerable cohort sample size (i.e., *n* = 45 patients) [21], as sample procurement can often be difficult to acquire for rare diseases. Additionally, their cohort consisted of patients who met TFC [15,16] for ACM (*n* = 35; 78%) and those who were gene carriers yet did not meet TFC (*n* = 10; 22%). The Editor applauds the authors for also stipulating certain limitations of their study. These included (i) the exact cellular and/or organismal origin of PICP and ICTP, (ii) the fact that blood collection and clinical data were not taken on the same day, and (iii) whether patients suffered from non-cardiac comorbidities associated with fibrotic remodeling (e.g., liver cirrhosis). However, one important limitation should be noted—the venous blood collected for this study utilized both sera and plasma samples [21]. While sera comprises a much larger portion of blood, it is more sensitive than plasma for the detection of biomarkers [31]. Yet, biomarker levels are more reproducible in plasma and are associated with a stronger predictive power than serum [31]. Lastly, plasma contains fibrinogen due to the presence of EDTA during venous collection, and thus, it additionally contains immunoglobulins and, inevitably, more protein [32].

To conclude, while circulating PICP and ICTP levels showed no correlation with the ECG parameters provided, a much larger study population may provide an assessment of whether PICP and ICTP levels correlate with the percentage of ectopic beats, bouts of non-sustained VT and sustained VT, and/or ICD discharges. Given the subtle but important differences between serum and plasma, these studies would undoubtedly provide more sensitivity and specificity if all venous blood was collected and processed identically. Notwithstanding, the findings by Van der Voorn and colleagues—in combination with those reported by Bacmeister L et al. [30]—indicate elevated levels of circulating PICP and ICTP and appear to be a reliable prognostic indicator of non-ischemic cardiac injury.
